# Phytochemical Characterization and In Vitro Antioxidant Properties of Four *Brassica* Wild Species from Italy

**DOI:** 10.3390/molecules25153495

**Published:** 2020-07-31

**Authors:** Valentina Picchi, Roberto Lo Scalzo, Aldo Tava, Filippo Doria, Sergio Argento, Stefania Toscano, Simone Treccarichi, Ferdinando Branca

**Affiliations:** 1 CREA Research Centre for Engineering and Agro-Food Processing, via G. Venezian 26, 20133 Milan, Italy; roberto.loscalzo@crea.gov.it; 2 CREA Research Centre for Animal Production and Aquaculture, viale Piacenza 29, 26900 Lodi, Italy; aldo.tava@crea.gov.it; 3 Department of Chemistry, University of Pavia, viale Taramelli 10, 27100 Pavia, Italy; filippo.doria@unipv.it; 4 CNR Istituto per i Sistemi Agricoli e Forestali del Mediterraneo (ISAFoM), via Empedocle 58, 95128 Catania, Italy; sergio.argento@cnr.it; 5 Dipartimento di Agricoltura, Alimentazione e Ambiente, Università degli Studi di Catania, via Valdisavoia 5, 95123 Catania, Italy; stefitosca@hotmail.it (S.T.); simone_t_95@hotmail.it (S.T.); fbranca@unict.it (F.B.)

**Keywords:** *Brassica* wild relatives, polyphenols, LC-UV-PDA-ESI-MS, DPPH, scavenging activity

## Abstract

In the present study, we evaluated for the first time the variability of antioxidant traits of four *Brassica* wild species: *B. incana*, *B. macrocarpa*, *B. villosa*, and *B. rupestris*. The content of the main water-soluble antioxidants (phenolics, ascorbic acid, and total biothiols) and the in vitro antioxidant potential (1,1-diphenyl-2-picrylhydrazil (DPPH) and superoxide anion scavenging capacity) were investigated. A total of 28 polyphenolic compounds were identified by LC/MS and quantitated by HPLC/DAD analysis. Kaempferol and quercetin derivatives were the most abundant phenolics compared to hydroxycinnamoyl gentiobiosides. In the ten populations, phenolics ranged from 163.9 to 533.9 mg/100 g dry weight (d.w.), ascorbic acid from 7.6 to 375.8 mg/100 g d.w., and total biothiols from 0.59 to 5.13 mg/100 g d.w. The different classes of phytochemicals were separated using solid-phase extraction at increasing methanol concentrations, and the antioxidant power of fractionated extracts was evaluated. The superoxide anion scavenging activity was significantly correlated to phenolics, particularly to flavonol derivatives, while DPPH was mainly related to ascorbic acid content. The present findings improve the knowledge of the phytochemical composition of Italian *Brassica* wild species by showing the great diversity of phytochemicals among populations and highlighting their importance as a valuable genetic resource for developing new cultivars with improved bioactive content.

## 1. Introduction

Within the broad genetic differences in *Brassica* genus, crop wild relatives (CWRs) are worth considering for their potential economic importance [1]. Wild species have survived and adapted to large historic climate variability and therefore may possess valuable genetic information for improving crop performance in the face of climate change [2]. Additionally, wild species have great potential as sources of bioactive compounds, because adaptation to challenging environments has led the plants to devote greater resources to synthesizing their own chemical defense mechanisms by increasing specialized secondary metabolites, including antioxidants [3].

In their work, Sánchez-Mata et al. [4] provided evidence that several wild and underutilized vegetables of the Mediterranean area showed a high content of bioactive compounds. In this context, *Brassicaceae* wild Mediterranean species could be of great interest as sources of nutraceuticals, such as glucosinolates and phenolics, and could be used in developing new cultivars with enhanced accumulation of phytochemicals and proposed as new types of health-promoting foods. In the remote past, Brassica CWRs intercrossed for several centuries in the Mediterranean basin. However, during the domestication process of several *B. oleracea* crops, the great variability of antioxidant traits was reduced. For this reason, Brassica CWRs were the subject of recent research aiming to evaluate and exploit their plasticity in traits [5].

In *Brassica*, phenolic compounds are widely considered to be the most important specialized metabolites with antioxidant activity, responsible for the beneficial effects on human health [6]. Hydroxycinnamic acids, such as caffeic, ferulic, sinapic, and *p*-coumaric acid, are among the most widespread phenolics in these plants, in which they are differently conjugated to organic acids, such as quinic and malic acid, and sugars, such as sophorose and gentiobiose [7,8,9]. Extensive studies on different varieties of *Brassica* species have disclosed variations in their composition [10,11,12]. Moreover, *Brassica* species are also rich in flavonols, with kaempferol, quercetin, and isorhamnetin as the main aglycones. They are mainly *O*-glycosylated at the 3- and 7-position of the C-ring of the aglycone with glucose, sophorose, or sophorotriose and often acylated with different hydroxycinnamic acids [13]. The compositional profile of flavonoids has been shown to vary among *Brassica* species [14,15,16,17,18].

The importance of consuming *Brassica* vegetables is also related to their high content of ascorbic acid (AsA) [7] as well as other water-soluble antioxidants, such as thiols [19,20,21]. Thiols are sulfur-containing compounds that have sulfhydryl functional groups (–SH), which play a crucial role in protecting cells from oxidative damage. Biologically derived thiols, such as glutathione, cysteine, and homocysteine, are often called biothiols and are present in plants at various concentrations. Limited and contrasting data exist about the content of biothiols in *Brassica* species. Demirkol et al. [19] reported a quantity of glutathione in broccoli of 0.2 mg/100 g fresh weight (fw), while Zacharis et al. [21] reported higher amounts in broccoli and Brussels sprouts (18.4 mg/100 g fw). On the other hand, Shigenaga et al. [20] revealed a reduced glutathione content of 30 mg/100 g fw in broccoli florets. All of the mentioned phytochemicals are important contributors of antioxidant potential, as reported in a number of studies carried out on *Brassica* species [6,18,22,23].

With the aim to preserve the agrobiodiversity of wild species, the *Brassica* Working Group of the European Cooperative Program for Genetic Resources (ECPGR), managed by Bioversity International, paid special attention to evaluating *Brassica* wild species (n = 9) including *B. incana*, *B. macrocarpa*, *B. rupestris*, and *B. villosa*, mainly grown in Southern Italy (Sicily) during the EU GENRES057 AEGRO project [24]. In a preliminary study, some wild *Brassica* species were examined for their glucosinolates, ascorbic acid, and total phenol and carotenoid content, including an evaluation of their antioxidant capacity [25].

As a continuation of a previous investigation [25], the present work was aimed at investigating the main water-soluble antioxidants (phenolics, ascorbic acid, and total biothiols) in ten populations of four *B. oleracea* wild species collected in Italy (*B. incana*, *B. macrocarpa*, *B. villosa*, *B. rupestris*) and to evaluate their antioxidant capacity in vitro. The relationships between the classes of phytochemicals and their antioxidant capacity are also presented and discussed.

## 2. Results and Discussion

### 2.1. Phenolic Content and Composition

**Figure 1:**
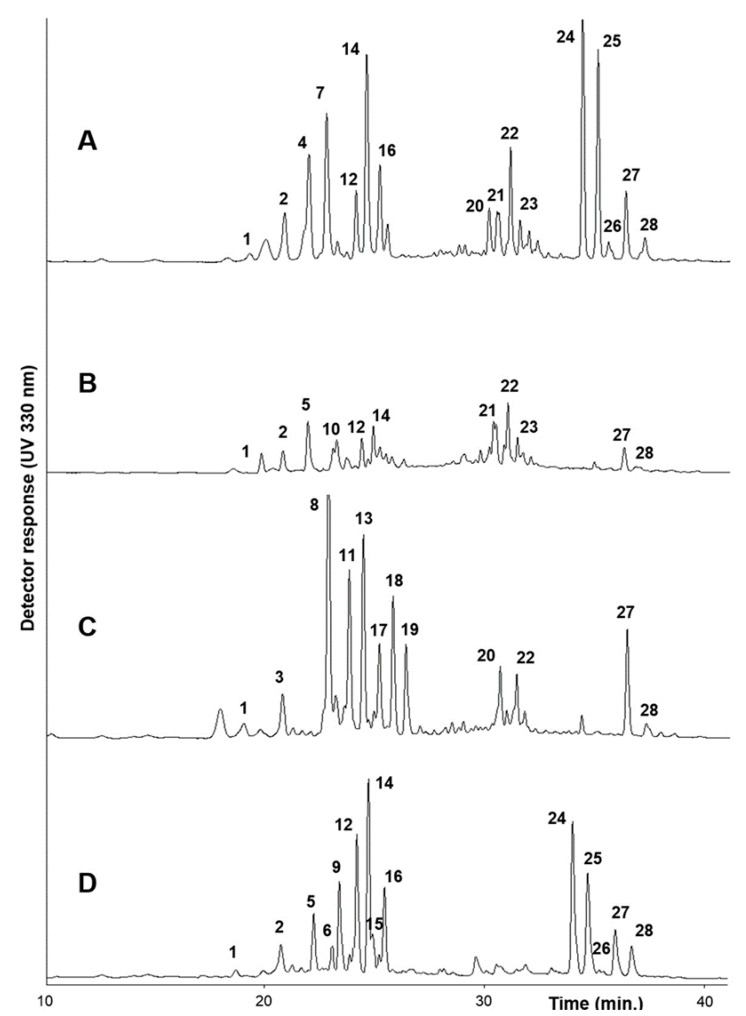
UPLC chromatogram (330 nm) of raw extracts of four Brassica species: (**A**) *B. incana*, (**B**) *B. macrocarpa*, (**C**) *B. rupestris*, and (**D**) *B. villosa*. For compound identification, see Table 1.

**Figure 2:**
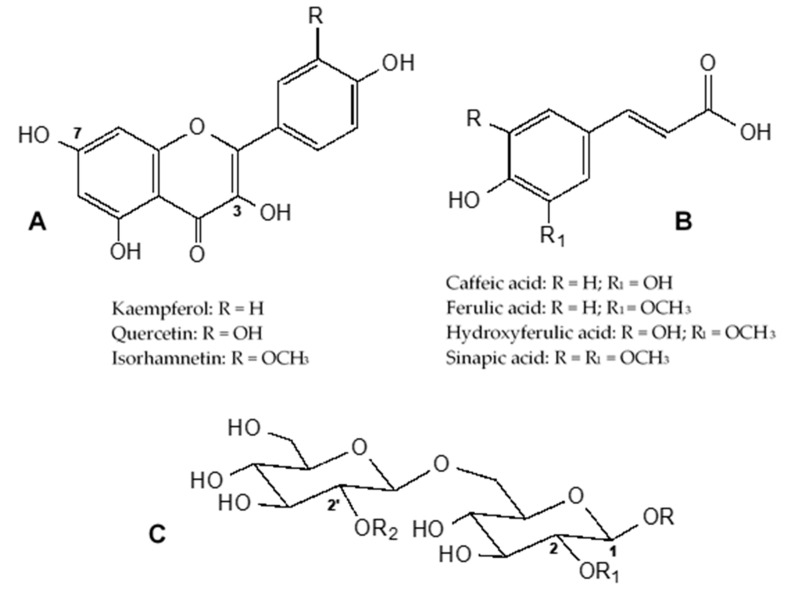
Chemical structure of phenolic compounds identified in wild Brassica samples: (**A**) flavonoids, (**B**) hydroxycinnamic acids, and (**C**) gentiobiosides.

The UPLC/DAD analysis showed the presence of a high number of phenolic compounds, as shown in Figure 1, and their UV spectra indicate that most of them were esters of hydroxycinnamic acid derivatives. All detected compounds are listed in Table 1. Their chemical structures are shown in Figure 2.

From the UPLC/MS/MS study, different groups of compounds were detected, including flavonoids, mostly represented by hydroxycinnamic acid esters of kaempferol and quercetin glycosides (compounds **1**–**23**), and hydroxycinnamic acyl glycosides, with a predominance of sinapoyl gentiobiosides (compounds **24**–**28**). Key fragment ions and other MS observations were also used for this evaluation. In positive ion mode, only the [M + H]^+^ ion was observed, while the [M − H]^−^ and MS^n^ ions were registered in the negative ion mode. For all detected compounds, a loss of 162 *m*/*z* was indicative of the presence of a hexose (glucose) and of 324 *m*/*z* the presence of a di-hexose (sophorose). Moreover, for hydroxycinnamic acid esters of flavonoid glycosides, losses of 162 *m*/*z*, 176 *m*/*z*, 176 *m*/*z*, and 206 *m*/*z* are also indicative of the presence of caffeic acid, ferulic acid, hydroxyferulic acid, and sinapic acid, respectively. For hydroxycinnamic acyl glycosides, a loss of 194 *m*/*z* is indicative of the presence of ferulic acid and of 224 *m*/*z*, the presence of sinapic acid [11,15,16,26,27,28,29]. As an example, the negative ion electrospray MS^2^ spectra of hydroxycinnamic acid esters of flavonoid glycosides **6**, **8**, and **16** are reported in Figure S1, and the negative ion electrospray MS^2^ spectra of hydroxycinnamoyl gentiobiosides **24**, **25**, and **28** are reported in Figure S2.

All compounds detected in the *Brassica* samples used in this investigation are listed in Table 1 and were previously identified and reported in several *Brassica* spp. [10,11,12,13,15,16,17,23,26,27,28,29,30,31]. Quantitative evaluation was performed by HPLC using an external standard method (see experimental section), and their amounts are also reported in Table 1. Great variability in their qualitative and quantitative composition can be seen. As a general trend, in all Brassica samples, kaempferol is the most detected flavonol compared to quercetin, and sinapoyl and feruloyl derivatives are the most abundant hydroxycinnamoyl esters compared to caffeoyl and hydroxyferuloyl derivatives.

Kaempferol synapoyl glucoside **14** (67.5–81.3 mg/100 g d.w.), kaempferol caffeoyl glucoside **7** (43.5–62.5 mg/100 g d.w.), and kaempferol feruloyl glucoside 4 (52.3–99.7 mg/100 g d.w.) were the most abundant flavonol derivatives in both the samples of *B. incana*. Kaempferol feruloyl glucoside **21** (30.7 ± 1.8 mg/100 g d.w.) and kaempferol glucosides **2** and **5** (23.7 ± 6.6 and 28.1 ± 1.9 mg/100 g d.w., respectively) were the most abundant compounds detected in *B. macrocarpa*.

*B. rupestris* extracts were characterized by a higher amount of quercetin sinapoyl glucoside **8** (12.5–73.7 mg/100 g d.w.), kaempferol feruloyl glucoside **11** (45.4–74.8 mg/100 g d.w.), and kaempferol synapoyl glucoside **13** (19.5–62.0 mg/100 g d.w.).

A higher amount of kaempferol synapoyl glucoside **12** (24.8–86.1 mg/100 g d.w.) and **14** (25.8–81.4 mg/100 g d.w.), quercetin sinapoyl glucoside **9** (11.6–76.1 mg/100 g d.w.), and compound **2**, kaempferol-3-*O*-diglucoside-7-*O*-diglucoside (46.9–80.4 mg/100 g d.w.), were the most abundant flavonol derivatives detected in *B. villosa* extracts.

A higher amount of hydroxycinnamoyl gentiobiosides (compounds **24**–**28**) was found in *B. incana* and *B. villosa* compared to *B. rupestris* and *B. macrocarpa*, in which only two hydroxycinnamoyl gentiobiosides were detected (compounds **27** and **28**). Among gentiobiosides, compound **24** (1,2-disinapoyl gentiobiose) and **25** (1-sinapoyl-2-feruloyl gentiobiose) were the most abundant in B. incana. In B. villosa, compound **27** (1,2,2′-trisinapoyl gentiobiose) was the most abundant in BL and DC, while compound **24** was predominant in BX. In BY5, we observed similar amounts of compounds **24**–**27** and a low content of compound **28**.

So far, no study has yet been carried out reporting the LC-MS characterization of the phenolic content of *Brassica* wild species, with the exception of *B. incana*, which was analyzed in recent work by Miceli et al. [31]. Compared to Miceli et al. [31], our extracts of *B. incana* contained more quercetin and kaempferol derivatives and five different hydroxycinnamoyl gentiobiosides, while isorhamnetin derivatives were not detected, indicating that different environmental growing conditions can affect the phenolic composition.

### 2.2. Quantification of Phytochemicals and their Fractionation

The content of water-soluble phytochemicals in raw extracts and fractions obtained after chromatographic separation of the 10 wild *Brassica* spp. under investigation (see experimental section) is reported in Table 2.

Figure S3 reports an example of the HPLC chromatograms of *B. rupestris* (BU15) raw extract (Figure S3A) and the two fractions, F50 (Figure S3B) and F80 (Figure S3C), obtained after chromatographic separation. These data confirmed good separation of the compounds and revealed that the F50 fraction was mostly constituted by flavonol glycosides (peaks **1**–**23**, Figure S3B), while the F80 fraction contained mostly hydroxycinnamoyl gentiobiosides (peaks **24**–**28**, Figure S3C). The F17 fraction did not contain any detectable phenol (data not shown); only ascorbic acid was found. Biothiols were detected only in raw extracts.

As reported in Table 2, *B. incana* samples showed the highest content of phenolics (531.0 ± 4.2 mg/100 g d.w. and 533.9 ± 23.0 mg/100 g d.w. in BN and BW, respectively) of which flavonol derivatives represent 70–80% of the total. *B. villosa* samples showed a quite constant phenolic content, in the range of 451.6–464.4 mg/100 g d.w., with flavonol derivatives representing 60–75% of the total. In raw extract of *B. rupestris*, phenolics ranged from 237.6 to 444.5 mg/100 g d.w., with about 90% of flavonol derivatives, while the *B. macrocarpa* sample showed the lowest phenolic content (163.9 ± 1.8 mg/100 g d.w.), of which flavonol derivatives represent 94% of the total. It is interesting to note that the wild *Brassica* species investigated here are characterized by a higher content of flavonol derivatives with respect to gentiobiosides, as shown in Table 2. It has been reported that in other *Brassica* species like broccoli, gentiobiosides were the most representative phenolic compounds [7,10].

Besides the importance of flavonols as powerful antioxidants [18], their health-related properties are also associated with their capacity to prevent AsA degradation by reverting the ascorbic radical to AsA and inhibiting the enzyme ascorbate oxidase [32].

The amounts of AsA and total thiols are also shown in Table 2. The highest AsA content was detected in *B. villosa* BX (375.8 mg/100 g d.w.) and in *B. incana* BN (355.7 mg/100 g d.w.). The same genotypes also showed the highest total thiol content, at 5.13 and 3.83 mg/100 g d.w., respectively. In BU5, BW, and BL, AsA ranged from 167.0 to 249.2 mg/100 g d.w., while BU9, BY5, and DC had the lowest AsA content (7.6–38.2 mg/100 g d.w.). BB, BY5, and DC were characterized by the lowest total thiol content, ranging from 0.59 to 0.83 mg/100 g d.w. Besides raw extracts, AsA was also detected in F17 fractions, although in lower amounts. The reduction of AsA content in F17 fractions was inversely related to its amount in the original raw extracts, with higher losses in samples that had the lowest AsA content in the raw extract, such as BY5 and DC (see Table 2).

*Brassica* crops have been intensively studied for their content of secondary metabolites, mainly phenolic compounds. Broccoli (*B. olearcea* L. *italica*) is also reported as a good source of ascorbic acid and thiols [33]. However, there is a lack of knowledge regarding the ascorbic acid and thiol content of *Brassica* wild species, and the present study attempts to fill this gap. The present findings show that *Brassica* wild species contained higher AsA and thiol content compared to other *Brassica* crops, such as broccoli [19,34], thus representing an importance source of bioactive compounds with potential positive effects on human health. In fact, AsA and thiols (particularly glutathione and cysteine) are widely recognized among the most effective antioxidants able to protect against various oxidative stress-related diseases like cancers cardiovascular disease and aging [34,35].

### 2.3. Antioxidant Capacity

**Figure 3:**
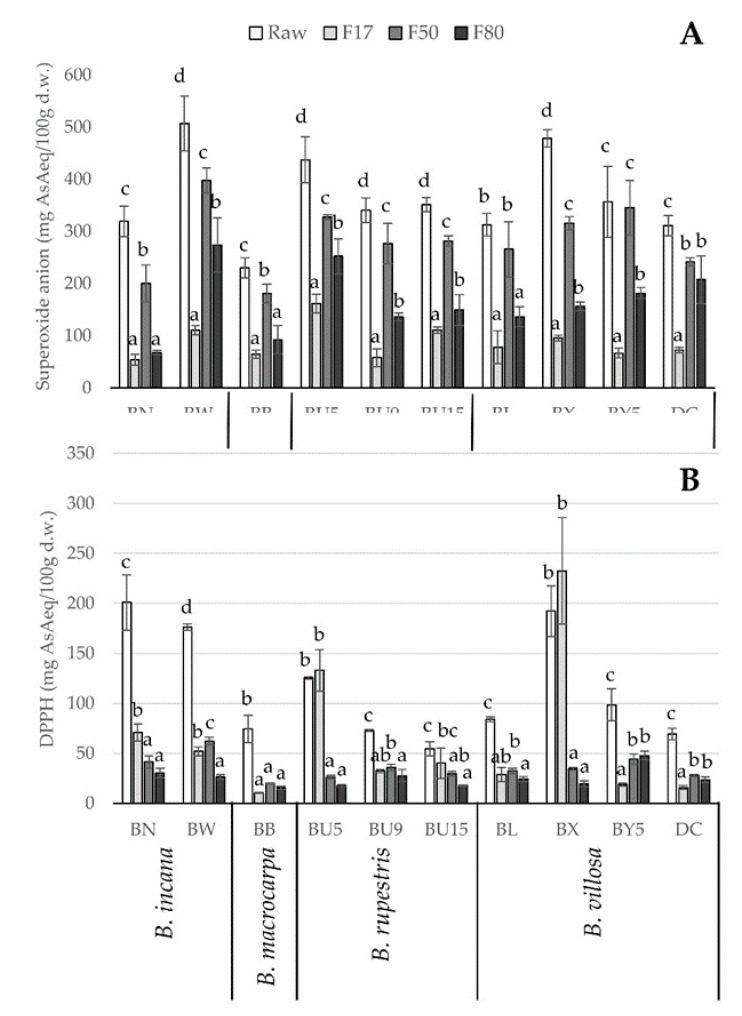
Antioxidant capacity of raw extracts and F17, F50, and F80 fractions of ten populations of *Brassica* wild species measured as (**A**) superoxide anion and (**B**) DPPH scavenging capacity. Letters above bars within each population indicate significantly different means according to Tukey’s test (*p* < 0.05).

Figure 3 shows the antioxidant capacity of the raw extracts and the three obtained fractions (F17, F50, and F80), evaluated by superoxide anion (Figure 3A) and DPPH (Figure 3B) methods.

With regard to the superoxide anion quenching capacity (Figure 3A), BW, BU5, and BX had the highest values (around 500 mg AsA eq/100 g d.w.), while other *Brassica* wild populations had slightly lower values (300–400 mg AsA eq/100 g d.w.). The BB sample showed the lowest superoxide scavenging capacity (250 mg AsA eq/100 g d.w.). The three fractions (F17, F50, and F80) contributed differently to the overall superoxide scavenging capacity, as shown in Figure 3A. F17, which only contains ascorbic acid, always had lower values with respect to F50 and, in most cases, F80, with the exception of three of the ten populations: BN, BB, and BL. These results indicate that phenolic compounds in F50 and F80 fractions were primarily responsible for the superoxide scavenging capacity. These results are in accordance with the study of Tabart et al. [36], which reported that the scavenging capacity against superoxide anion was strongly related to phenolic compounds. Indeed, the superoxide scavenging activity of raw extracts was significantly correlated with the total phenolic content (r = 0.53, *p* < 0.001) and particularly with flavonol derivatives (r = 0.58, *p* < 0.001), which were mainly retained in the F50 fraction. In fact, the superoxide scavenging activity of raw extracts was significantly correlated to the scavenging capacity of the F50 fraction (r = 0.77, *p* < 0.001), while the F17 and F80 fractions were less correlated (r = 0.59 for both fractions, *p* < 0.05).

Some authors studied the influence of glycosylated and acylated flavonol derivatives on the antioxidant activity of kale (*B. oleracea* var. *sabellica*) and showed that flavonol derivatives possessed higher antioxidant capacity than gentiobiosides [17,37]. Moreover, among flavonols, quercetin and its derivatives seem to be more capable of scavenging free radicals than kaempferol, because of the catechol structure of the B-ring [38,39]. This finding explains the higher superoxide scavenging capacity of the F50 fraction, which contained mainly flavonol derivatives.

The results of DPPH scavenging capacity of the raw extracts and the three obtained fractions (F17, F50, and F80) are shown at the bottom of Figure 3. The raw extracts of genotypes BN, BW, and BX showed the highest values (around 200 mg AsA eq/100 g d.w.), whereas the other populations ranged from 54.7 (BU15) to 125.4 (BU5) mg AsA eq/100 g d.w. The DPPH scavenging capacity of raw samples was primarily dependent on AsA content (r = 0.82, *p* < 0.001) and thiols (r = 0.76, *p* < 0.001), whereas flavonol derivatives contributed to a lesser extent (r = 0.35, *p* < 0.05). With regard to the contributions of single fractions to total DPPH scavenging capacity, Figure 3B shows that F17 had higher DPPH values compared to F50 and F80 in BN, BU5, and BX. In the other populations, the DPPH scavenging capacity of the three fractions was similar. In BY5 and DC, which were distinguished by the lowest AsA content (Table 2), the DPPH values were lower in F17 than in F50 and F80. These results suggest a strong dependence of DPPH scavenging capacity on AsA, which indeed characterized the F17 fraction. The primary role of AsA in determining DPPH scavenging capacity was already observed in other *Brassica* extracts [10,40]. In line with this observation, linear regression analysis showed a good relationship between DPPH values of raw extract and F17 fraction (r = 0.67, *p* < 0.0001), while a lower correlation was found with F50 fraction (r = 0.38, *p* < 0.05). These findings are in line with the rationale that AsA is the fastest scavenger of DPPH, as it reacts rapidly with DPPH, reaching a steady state in less than 1 min [41], corresponding to the reaction time used in the present work.

As a whole, the results of antioxidant capacity determined by the two tests highlight that *Brassica* species with high phenolic and AsA content, as in the case of BN, BW, BU5, and BX, are the ones with the greatest potential positive effects on human health.

**Figure 4:**
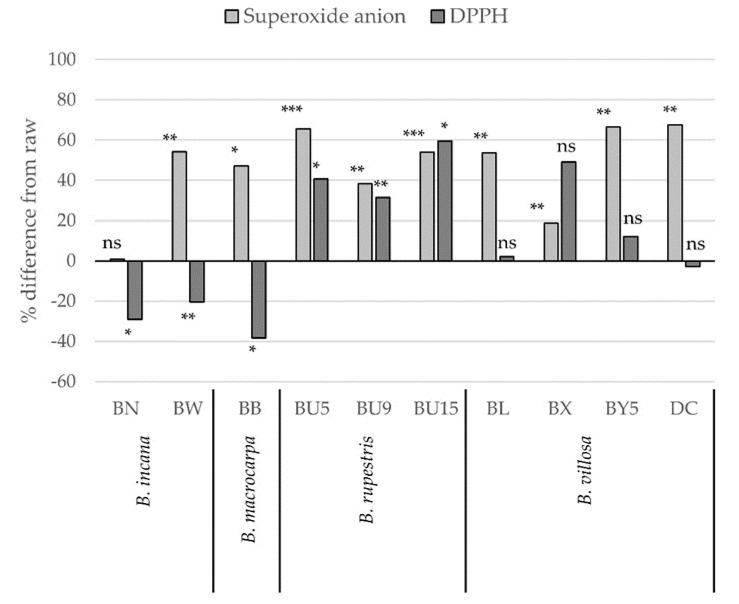
Percent differences with respect to raw extracts (assumed equal to 100%) of superoxide anion and DPPH^•^ scavenging activity obtained by the sum of values of three fractions (F17, F50, F80) in ten *Brassica* wild species. * *p* < 0.05, ** *p* < 0.01, *** *p* < 0.001, ns, *p* > 0.05, according to Tukey’s test.

Figure 4 shows the percent differences with respect to raw extracts (assumed equal to 100%) of superoxide anion and DPPH scavenging activity obtained by the sum of the values of the three fractions (F17, F50, and F80) in the ten *Brassica* wild species. Summing the antioxidant capacity values of each fraction, we observed that in most cases, the superoxide and DPPH scavenging capacity was significantly higher compared to raw extracts. Only in the case of *B. incana* (BN and BW) and *B. macrocarpa* (BB), the lower DPPH scavenging capacity based on the sum of the three fractions was probably related to the loss of AsA during the fractionation process (around 40–50% of the total; see Table 2) and the high dependency of DPPH scavenging capacity on AsA content itself. Since the levels of antioxidant metabolites (AsA, thiols, and phenols) were obviously higher in raw samples compared to the level obtained by summing the content in each fraction (see Table 2), we can hypothesize a form of antagonism among the phytochemicals of single fractions when mixed. Indeed, it is difficult to establish a clear relationship between the antioxidant content in a complex matrix and the antioxidant activity of a single compound. For example, while Marin et al. [32] observed a positive effect of flavonoid on ascorbic acid regeneration, other authors [42] showed that quercetin may act as a pro-oxidant in the presence of thiols, possibly because of an induced increase of reactive oxygen species and the formation of harmful thiol radicals. Experiments with different antioxidant assays and model solutions with mixed antioxidants gave extremely different responses in terms of antagonism, synergism, and additive effect [43,44,45].

The results of the multilinear regression (MLR) analysis indicated that the concentration of phenolics and AsA, but not thiols, could be a good predictor of DPPH scavenging capacity, since the total variance explained by the model was 69% (Figure S4). By contrast, the same was not true for the superoxide scavenging capacity, where the total variance explained by the model was equal to 24% (data not shown). The correlation between predicted and measured values of DPPH showed highly significant values with a coefficient of determination (R^2^) of 0.799 in calibration (Cal) and 0.693 in cross-validation (CV). Low values and results of MLR prediction were found for superoxide scavenging activity (R^2^ Cal = 0.416; R^2^ CV = 0.239). These findings confirm the strong dependence of DPPH scavenging capacity on AsA content and suggest the need to analyze not only phenols but also AsA when determining the total antioxidant capacity of a complex matrix.

## 3. Materials and Methods

### 3.1. Plant Material

Seeds of wild populations of *B. incana* (Figures S5.1 and S5.2), *B. macrocarpa* (Figures S5.3 and S5.4), *B. rupestris* (Figures S5.5 and S5.6), and *B. villosa* (Figures S5.7 and S5.8) belonging to the *B. oleracea* complex species (number of chromosomes = 9) were collected in different areas of Sicily during AEGRO projects in 2006–2010. *B. incana* is mainly present along the eastern and northeastern Sicilian coast, *B. macrocarpa* is an endemic species of the Egadi Islands, *B. rupestris* is widespread along the northern coast of Sicily, mainly in Western areas, and *B. villosa* grows in the central-western inland areas and along the northwestern coast. In addition, one population of *B. incana* and one of *B. rupestris* collected in the Lazio and Calabria regions, respectively, were also investigated. Voucher specimens of each population are maintained at the Department of Agriculture, Food and Environment of the University of Catania. The complete list of accessions is shown in Table 3. *Brassica* populations were grown in the experimental farm of the University of Catania (37°27′ N, 15°40′ E, 10 m a.s.l.). Eight months after transplanting, leaf samples were collected and immediately frozen and lyophilized. The freeze-dried samples were ground to a fine powder at low temperature and stored at –20 °C.

### 3.2. Extraction and Fractionation

Sample extraction and fractionation were performed according to Rodríguex et al. [46], with modifications. The raw extract was obtained by adding 1 g of lyophilized powder to 15 mL of MeOH/0.03 N HCl (1:1). The mixture was shaken at room temperature for two hours and then centrifuged at 25,000× *g* at 4 °C for 20 min. The supernatant was filtered on glass wool and stored at −80 °C. Fractionation of raw extracts was performed on 1.2 cm diameter cartridges filled with 2 g of ICN Biomedicals C18 adsorbent resin (32–63 µm, 60 A) preconditioned with 3 × 5 mL of 0.03 N HCl. The sample (4 mL) was diluted three-fold with 0.03 N HCl and then loaded at a flow rate of 1 mL/min. The sample was eluted with 4 mL of three solutions at increasing MeOH concentration, thus obtaining three fractions: (1) F17, eluted with 17% MeOH in 0.03 N HCl; (2) F50, eluted with 50% MeOH in 0.03 N HCl; and (3) F80, eluted with 80% MeOH in 0.03 N HCl.

Further elution with increasing MeOH up to 100% did not reveal any other compound, as confirmed by HPLC analysis. The three collected fractions were then stored at –80 °C until analysis. Each sample was analyzed in triplicate.

### 3.3. LC/UV–DAD/ESI–MS Analysis

LC-MS analysis was performed on raw extracts using a Jasco UPLC-system combined with a Thermo LTQ. A Thermo Xcalibur Qual Browser was used for the acquisition and processing of mass data. The UV-vis chromatograms were analyzed with a Jasco Chemstation (ChromNAV, Jasco, Mary’s Court, Easton, MD, USA). Chromatographic analysis was carried out on an Acquity UPLC BEH C18 column (1.7 µm; 50 × 2.1 mm, Phenomenex, Torrence, CA, USA). All analyzed samples were dissolved in MeOH/H_2_O (9:1) at a concentration of 20–30 ppm. The column was set at 35 °C. The mobile phase for UPLC analysis was 0.1% formic acid in water and acetonitrile. The samples were spectrophotometrically checked by reading at eight wavelengths (205 nm to 275 nm). Elution was performed with a flow rate of 0.3 mL/min according to the following gradient method: injection volume 5 µL; linear gradient starting from 95% aqueous, gradually to 25% of acetonitrile over 30 min, gradually to 70% of acetonitrile within 40 min, gradually to 100% of acetonitrile over 50 min, and then isocratic flow for 5 min. Mass spectrometry data were acquired in positive and negative ion mode. All analyses were carried out using an ESI ion source with the following settings: capillary voltage, 3100 V; sheath gas (He), aux gas (He), and sweep gas (He) heated at 275 °C and introduced with a source heater temperature of 80 °C. Full scan spectra were acquired over the range of 100–2000 *m*/*z*. Automated MS/MS was performed by isolating the base peaks (molecular ions) using an isolation width of 2.0 *m*/*z*, normalized collision energy of 25 V, threshold set at 500, and ion charge control on, with max acquisition time set at 300 ms. All compounds were tentatively identified by comparing their retention times and mass spectral data obtained in positive and negative ion mode with those of standard compounds or with compounds previously reported in the literature for *Brassica* spp. [11,15,16,26,27,28,29,30].

### 3.4. HPLC-DAD Analyses

The phenolic composition of raw and fractionated extracts was also evaluated by HPLC-DAD using a Jasco system equipped with a diode array detector (MD-910, Jasco, Mary’s Court, Easton, MD, USA). The pump (PU-980, Jasco, Mary’s Court, Easton, MD, USA) was coupled with a ternary gradient unit (LG-1580-02, Jasco, Mary’s Court, Easton, MD, USA). The analytical data were evaluated using a software management system for chromatographic data (ChromNAV, Jasco, Mary’s Court, Easton, MD, USA). The separation was performed by a reversed phase C18 Purospher Star column (250 × 4 mm, 5 µ, Merck, Darmstadt, Germany) was coupled with a ternary gradient unit (LG-1580-02, Jasco, Mary’s Court, Easton, MD, USA). The flow rate was 0.6 mL/min, the injection volume was 10 μL, and the column temperature was maintained at 42 °C. The mobile phase consisted of water with 5% of glacial acetic acid (solvent A) and acetonitrile with 5% of glacial acetic acid (solvent B). The gradients were as follows (A/B): 95/5 from 0–10 min, 95/5 to 85/15 in 10 min, 85/15 to 70/30 in 10 min, 70/3 for 10 min, 70/30 to 95/5 in 10 min, and 95/5 for 10 min. Peak identification was based on LC-MS data. Quantitation of detected compounds was performed at 330 nm by an external standard method using available commercial standards (purity HPLC ≥98%) of kaempferol-3-*O*-glucoside (y = 134,103x – 103,094, R^2^ = 0.9942) and quercetin-3-*O*-glucoside (y = 112,896x + 58,728, R^2^ = 0.9959) for flavonol derivatives (peaks **1**–**23**) and sinapic acid acyl-β-d-glucoside (y = 194,026x + 13,9401, R^2^ = 0.9960) for hydroxycinnamoyl gentiobiosides (peaks **24**–**28**). For calibration curves, all standards were injected in triplicate in the range of 4 to 40 µg/mL.

### 3.5. Ascorbic Acid Analysis

Ascorbic acid was analyzed in raw extracts and in the three fractions following the method described by Picchi et al. [47], with slight modifications. For sample preparation, 200 µL of extract was treated with 800 µL of cold 3% metaphosphoric acid, homogenized, centrifuged at 25,000× *g* for 5 min at 4 °C, and immediately analyzed. The analytical column was a 250 × 6 mm, 5 µ Intersil ODS-3, maintained at 30 °C. The isocratic elution was performed using orthophosphoric acid 0.02 M as mobile phase, at a flow rate of 0.7 mL/min. Samples of 20 μL were injected and monitored at 254 nm. The concentration of ascorbic acid was calculated from the experimental peak area by analytical interpolation in a standard calibration curve (range 1.0–100.0 mg/100 mL) and was expressed as mg/100 g dry weight (d.w.). Ascorbic acid was detected in raw extracts and in F17 fractions, while it was not found in F50 and F80 fractions.

### 3.6. Analysis of Thiol Groups

The determination of non-protein –SH groups in the extracts and fractions was performed according to Hawrylak and Szymanska [48], with some modifications. An aliquot of 0.1 mL of sample was added to 0.8 mL of 1 M sodium phosphate buffer (pH 8.0) and 0.1 mL of 10 mM dithionitrobenzoic acid (DTNB). The absorbance was then read at 415 nm. The amount of biothiols was calculated from a standard curve made for l-cysteine, and the data are reported as mg/100 g d.w. Total thiol groups were only detected in raw extracts.

### 3.7. Antioxidant Capacity Measurement

The antioxidant capacity potential was measured using 1,1-diphenyl-2-picrylhydrazil (DPPH) radical quenching and superoxide anion radical scavenging. All measurements were performed by electronic paramagnetic resonance (EPR) using a MiniScope MS200 (Magnettech, Berlin, Germany). The protocol was detailed in Picchi et al. [47]. The calibration was made with ascorbic acid solution at known concentration and the results were reported as equivalent of ascorbic acid/100 g d.w.

### 3.8. Statistical Analysis

The analyses were carried out using three biological replicates. The phytochemical data of raw extracts were subjected to one-way analysis of variance and comparisons among means were performed according to the least significant difference (LSD) test. For each species, the antioxidant activity of the four extracts (raw, F17, F50, F80) was compared according to the LSD test. Significant differences were accepted at the minimum probability level of *p* < 0.05. A multilinear regression (MLR) analysis was carried out using the data of raw extracts to assess a possible relationship between the concentrations of all antioxidants analyzed in this paper (flavonol derivatives, hydroxycinnamoyl gentiobiosides, ascorbic acid, and thiols) and their overall antioxidant capacity, as measured using the DPPH and superoxide scavenging test. All measured antioxidants (independent variables) were considered in the statistical development of the MLR model. The procedure included the following steps: (1) extraction of raw data of antioxidants (X block variables); (2) extraction of measured values of antioxidant activity (DPPH or superoxide scavenging activity, Y block variables); (3) application of preprocessing algorithms to X and Y; (4) application of the MLR multivariate technique for modelling and testing; and (5) calculation of efficiency parameter of prediction. The stability and validity of the model were tested using Venetian blind cross-validation. For variable selection, a stepwise procedure was utilized. The model includes a calibration phase and a validation phase, with residual errors calculated for both root mean square error in calibration (RMSEC) and cross-validation (RMSECV). The predictive ability of the model was tested with the coefficient of determination (R^2^) between observed and predicted values.

## 4. Conclusions

The ten Mediterranean wild relatives of *B. oleracea* considered here were evaluated for the first time for their content of main water-soluble antioxidants, namely ascorbic acid, phenolics, and total biothiols. The results showed great variability in terms of the qualitative and quantitative composition of these metabolites. LC/MS investigation of polyphenolics showed that the greatest constituents were represented by hydroxycinnamoyl glycosides of the flavonols kaempferol and quercetin and by hydroxycinnamoyl gentiobiosides. The evaluation of the in vitro antioxidant activity on fractionated extracts contributes to clarifying the relationship between bioactive compounds of *Brassica* wild species and their antioxidant capacity. The results highlight the importance of consuming *Brassica* wild species because of their high content of flavonol derivatives, ascorbic acid, and thiols with respect to commonly cultivated *Brassica* genotypes. These metabolites are known to be very powerful antioxidants, indicating that consuming *Brassica* wild species may have an important impact on human health. Besides the great diversity of phytochemicals among populations and their good antioxidant potential, the Italian *Brassica* wild relatives may represent a valuable genetic source for developing new cultivars with improved bioactive metabolite content. Future work aimed at studying the in vivo antioxidant potential of *Brassica* wild species could help to confirm the high nutritional value of these edible plants and recognize their importance in the human diet for their health properties because of the capacity of effectively keeping under control the production of reactive oxygen species.

## Figures and Tables

**Table 1:** Content (mg/100 g d.w.) and composition of tentatively identified polyphenols in wild *Brassica* spp. used in this investigation.

#	UV λ_max_ (nm)	[M + H]^+^/ [M − H]^-^	MS^2^ (*m*/*z*) (%)	Compound ^a^	*B.incana*			*B. macrocarpa*		*B. rupestris*				*B. villosa*			
					BN	BW		BB		BU5	BU9	BU15		BL	BX	BY5	DC
**1**	235, 263, 319	951/949	625(100); 301(5)	Q-3-*O*-diglucoside- 7-*O*-diglucoside	2.3 ± 0.0	9.6 ± 0.1		18.1 ± 0.1		6.0 ± 0.4	69.2 ± 10.7	2.5 ± 0.02		13.0 ± 0.2	3.6 ± 0.3	5.7 ± 0.6	6.6 ± 0.0
**2**	239, 263, 338	935/933	701(100); 285(2)	K-3-*O*-triglucoside- 7-*O*-glucoside	51.2 ± 1.9	3.3 ± 0.5		23.7 ± 6.6		-	-	-		50.2 ± 3.1	46.9 ± 2.7	80.4 ± 8.6	66.9 ± 3.1
**3**	237, 255, 330	1275/1273	1111(100); 949(43); 787(68); 301(1)	Q-3-*O*-caffeoyltriglucoside- 7-*O*-diglucoside	-	-		-		15.1 ± 1.1	59.8 ± 9.1	18.8 ± 7.4		-	-	-	-
**4**	239, 267, 331	1127/1125	801(100); 609(27); 285(1)	K-3-*O*-hydroxyferuloyl diglucoside-7-*O*-diglucoside	52.3 ± 1.9	99.7 ± 1.3		-		-	-	-		-	-	-	-
**5**	235, 263, 330	935/933	609(100);285(2)	K-3-*O*-diglucoside- 7-*O*-diglucoside	-	-		28.1 ± 1.9		-	-	-		52.9 ± 3.0	36.0 ± 2.2	16.3 ± 2.0	50.1 ± 1.3
**6**	239, 267, 331	995/993	831(100); 787(88); 625(56); 301(1)	Q-3-*O*-sinapoyldiglucoside- 7-*O*-glucoside	-	-		-		-	-	-		16.5 ± 0.5	9.1 ± 1.2	21.2 ± 2.1	30.7 ± 0.1
**7**	243, 267, 331	1097/1095	771(100); 609(17)	K-3-*O*-caffeoyldiglucoside- 7-*O*-diglucoside	62.5 ± 2.1	43.5 ± 2.2		-		-	-	-		-	-	-	-
**8**	245, 340	1319/1317	1111(100); 993(72); 787(87); 301(2)	Q-3-*O*-sinapoyltriglucoside- 7-*O*-diglucoside	-	-		-		54.0 ± 2.3	73.7 ± 9.6	12.5 ± 0.0		-	-	-	-
**9**	237, 339	1157/1155	949(100); 831(32); 625(35); 301(1)	Q-3-*O*-sinapoyldiglucoside- 7-*O*-diglucoside	-	-		-		-	-	-		76.1 ± 2.4	38.9 ± 2.4	17.7 ± 2.6	11.6 ± 0.5
**10**	235, 340	951/949	787(100); 625(52); 301(18)	Q-3-*O*-caffeoyldiglucoside- 7-*O*-glucoside	-	-		9.2 ± 0.6		-	-	-		-	-	-	-
**11**	253, 347	1289/1287	1111(93); 963(75); 787(100); 301(1)	K-3-*O*-feruloyltriglucoside- 7-*O*-diglucoside	-	-		-		45.4 ± 12.3	62.1 ± 9.3	74.8 ± 0.2		-	-	-	-
**12**	239, 267, 331	979/977	815(100); 771(2); 609(7); 285(2)	K-3-*O*-sinapoyldiglucoside- 7-*O*-glucoside	27.7 ± 0.3	28.2 ± 0.7		7.0 ± 0.1		-	-	-		40.0 ± 1.3	86.1 ± 6.1	24.8 ± 3.7	31.3 ± 0.6
**13**	239, 267, 330	1303/1301	1095(7); 977(100); 771(75); 285(2)	K-3-*O*-sinapoyltriglucoside- 7-*O*-diglucoside	-	-		-		62.0 ± 2.2	19.5 ± 2.9	36.7 ± 0.4		-	-	-	-
**14**	243, 267, 331	1141/1139	815(100); 609(28); 285(1)	K-3-*O*-sinapoyldiglucoside- 7-*O*-diglucoside	81.3 ± 1.8	67.5 ± 0.7		7.7 ± 0.4		-	-	-		27.1 ± 0.9	81.4 ± 4.4	37.1 ± 3.9	25.8 ± 1.3
**15**	239, 264, 331	949/947	785(100); 609(7); 285(2)	K-3-*O*-feruloyldiglucoside- 7-*O*-glucoside	-	-		-		-	-	-		21.5 ± 0.6	10.1 ± 1.2	43.3 ± 4.2	32.1 ± 0.1
**16**	239, 267, 331	1111/1109	785(100); 609(21); 285(1)	K-3-*O*-feruloyldiglucoside- 7-*O*-diglucoside	38.5 ± 1.1	25.3 ± 1.5		-		-	-	-		34.3 ± 1.5	30.9 ± 1.9	30.6 ± 3.0	42.3 ± 0.2
**17**	239, 265, 332	1273/1271	1095(10); 947(100); 771(64); 285(3)	K-3-*O*-feruloyltriglucoside- 7-*O*-diglucoside	-	-		-		19.5 ± 0.1	26.2 ± 4.0	16.7 ± 0.1		-	-	-	-
**18**	239, 263, 343	935/933	771(100); 609(10); 285(5)	K-3-*O*-caffeoyldiglucoside- 7-*O*-glucoside	-	-		-		29.4 ± 0.3	39.0 ± 5.8	18.6 ± 0.3		-	-	-	-
**19**	239, 253, 353	965/963	801(100); 625(9); 301(3)	Q-3-*O*-feruloyldiglucoside- 7-*O*-glucoside	-	-		-		12.6 ± 0.1	31.9 ± 4.8	10.6 ± 0.2		-	-	-	-
**20**	239, 269 sh, 329	1495/1493	1169(100); 609(39); 285(7)	K-3-*O*-sinapoylhydroxyferuloyl triglucoside-7-*O*-diglucoside	10.4 ± 0.3	20.0 ± 2.1		-		19.1 ± 0.2	20.0 ± 2.9	11.1 ± 0.0		-	-	-	-
**21**	239, 269 sh, 330	1465/1463	1139(100); 609(21); 285(3)	K-3-*O*-feruloylhydroxyferuloyl triglucoside-7-*O*-diglucoside	5.6 ± 0.2	31.3 ± 2.7		30.7 ± 1.8		-	-	-		-	-	-	-
**22**	239, 267, 331	1509/1507	1301(7); 1183(100); 609(27); 285(5)	K-3-*O*-disinapoyltriglucoside- 7-*O*-diglucoside	22.4 ± 1.3	53.0 ± 2.7		18.2 ± 1.5		18.9 ± 0.3	24.0 ± 3.8	16.2 ± 1.8		-	-	-	-
**23**	239, 267 sh, 327	1479/1477	1153(100); 947(25); 609(33); 285(4)	K-3-*O*-feruloylsinapoyl triglucoside-7-*O*-diglucoside	16.6 ± 0.4	37.7 ± 3.0		10.9 ± 1.3		-	-	-		-	-	-	-
**24**	240, 331	777 ^b^/753	529(100)	1,2-disinapoyl gentiobioside	57.2 ± 0.0	51.3 ± 2.4		-		-	-	-		27.8 ± 0.7	50.6 ± 2.4	39.4 ± 3.7	17.1 ± 0.2
**25**	240, 327	747 ^b^/723	529(38); 499(100)	1-sinapoyl-2-feruloyl gentiobioside	55.6 ± 0.7	55.6 ± 1.3		-		-	-	-		15.5 ± 0.1	36.9 ± 3.6	48.7 ± 3.9	33.3 ± 0.7
**26**	239, 294 sh, 327	717 ^b^/693	499(100)	1,2-diferuloyl gentiobioside	9.0 ± 0.0	1.6 ± 0.8		-		-	-	-		0.94 ± 0.0	0.7 ± 0.0	45.4 ± 4.2	32.4 ± 0.8
**27**	240, 327	983 ^b^/959	735(100); 511(21)	1,2,2′-trisinapoyl gentiobioside	20.9 ± 0.7	5.4 ± 1.2		8.9 ± 0.7		34.5 ± 0.5	28.1 ± 4.0	18.3 ± 0.3		55.1 ± 3.2	19.5 ± 0.9	45.7 ± 4.2	66.3 ± 1.8
**28**	239, 327	953 ^b^/929	735(98); 705(100); 511(13); 481(7)	1,2′-disinapoyl-2-feruloyl gentiobioside	14.8 ± 0.1	0.98 ± 0.0		1.3 ± 0.1		3.4 ± 0.04	7.6 ± 1.1	0.75 ± 0.0		33.7 ± 0.5	12.8 ± 2.5	8.3 ± 0.7	5.2 ± 0.5

^a^K, kaempferol; Q, quercetin. ^b^[M + Na]^+^ for gentiobiosides.

**Table 2:** Content of total water-soluble antioxidants (mg/100 g d.w.) of wild *Brassica* spp. Flavonol derivatives, hydroxycinnamoyl derivatives, and total phenols were detected in raw, F50, and F80 extracts. Ascorbic acid (AsA) was detected in raw and F17. Biothiols were present only in raw extracts.

		*B.incana*			*B. macrocarpa*		*B. rupestris*				*B. villosa*			
		BN	BW		BB		BU5	BU9	BU15		BL	BX	BY5	DC
Flavonol	*Raw*	370.6 ± 6.8 g	419.0 ± 17.2 h		153.7 ± 0.9 a		282.0 ± 13.7 c	430.2 ± 30.2 h	218.6 ± 10.5 b		325.5 ± 6.1 de	343.0 ± 22.4 ef	277.0 ± 61.5 c	297.4 ± 5.7 cd
derivatives	*F50*	284.6 ± 38.1	275.1 ± 5.0		109.1 ± 6.4		262.1 ± 10.7	152.8 ± 21.0	166.9 ± 2.6		344.3 ± 17.6	338.4 ± 10.5	101.3 ± 19.2	159.0 ± 19.2
	*F80*	n.d.	69.9 ± 7.7		27.3 ± 2.7		33.2 ± 2.1	33.5 ± 4.2	n.d.		57.5 ± 17.0	38.7 ± 6.8	76.2 ± 16.4	8.3 ± 0.3
Hydroxy-	*Raw*	157.4 ± 1.5 e	114.9 ± 5.8 c		10.2 ± 0.8 a		38.0 ± 13.7 b	37.5 ± 2.6 b	19.0 ± 0.3 a		133.0 ± 4.5 d	120.5 ± 9.4 cd	187.4 ± 33.4 f	154.2 ± 2.6 e
cinnamoyl	*F50*	9.3 ± 0.3	21.6 ± 1.8		1.7 ± 0.2		3.2 ± 0.4	8.8 ± 0.8	1.0 ± 0.2		10.7 ± 2.5	10.2 ± 2.2	24.8 ± 2.0	18.1 ± 8.0
gentiobiosides	*F80*	113.0 ± 13.3	93.7 ± 7.3		8.9 ± 0.6		32.4 ± 0.2	51.6 ± 7.7	18.3 ± 0.6		91.1 ± 4.2	121.0 ± 3.0	181.0 ± 20.4	116.4 ± 4.2
Total phenols	*Raw*	531.0 ± 4.2 e	533.9 ± 23.0 e		163.9 ± 1.8 a		319.9 ± 13.2 c	444.5 ± 65.6 d	237.6 ± 10.7 b		454.7 ± 5.3 d	463.5 ± 31.8 d	464.4 ± 94.9 d	451.6 ± 8.3 d
AsA	*Raw*	355.7 ± 60.8 f	249.2 ± 42.6 e		103.9 ± 17.8 b		214.8 ± 36.7 de	30.1 ± 12.2 a	119.8 ± 20.5 bc		167.0 ± 28.5 cd	375.8 ± 64.2 f	7.6 ± 1.3 a	38.2 ± 6.5 a
	*F17*	122.8 ± 3.5	96.5 ± 3.9		37.0 ± 10.5		101.5 ± 6.5	14.6 ± 0.2	70.0 ± 7.2		76.4 ± 21.9	131.0 ± 9.1	1.1 ± 0.1	5.7 ± 2.1
	%*	- 65.5	- 61.3		- 64.4		- 52.8	- 51.6	- 41.6		- 54.2	- 65.1	- 85.2	- 85.1
Biothiols	*Raw*	3.83 ± 1.02 d	1.88 ± 0.5 c		0.59 ± 0.16 a		1.19 ± 0.45 abc	1.7 ± 0.0 bc	1.02 ± 0.27 abc		0.89 ± 0.24 abc	5.13 ± 1.37 e	0.67 ± 0.18 ab	0.83 ± 0.22 ab

Values are reported as mean ± standard deviation. Significantly different means in a row are indicated with different letters (*p* < 0.001). %* indicates percent reduction of AsA in F17 compared to raw extract.

**Table 3:** List of wild *Brassica* populations evaluated in this work.

Species	Code	Origin	Coordinates	Accession Code
*B. incana*	BN	Agnone Bagni (SR)	37°19′ N 15°05′ E	UNICT4419
	BW	Sortino (SR)	37°10′ N 15°20′ E	UNICT4158
*B. macrocarpa*	BB	Favignana (TP)	37°55′ N 12°19′ E	UNICT4801
*B. rupestris*	BU5	Roccella Valdemone (ME)	37°56′ N 15°10′ E	UNICT3405
	BU9	Ragusa Ibla (RG)	36°56′ N 14°45′ E	UNICT3458
	BU15	Stilo (RC)	38°29′ N 16°28′ E	UNICT3411
*B. villosa*	BL	Caltabellotta (AG)	37°34′ N 13°13′ E	UNICT5031
	BX	Marianopoli (CL)	37°36′ N 13°55′ E	UNICT3944
	BY5	Agnone Bagni (SR)	37°19′ N 15°05′ E	UNICT4581
	DC	Lago Albano (RM)	41°45′ N12°39′ E	UNICT4202

## Supplementary Materials

The following are available online. Figure S1: Negative ion electrospray MS2 mass spectra of hydroxycinnamic acid esters of flavonol glycosides: (A) quercetin 3-O-sinapoyldiglucoside-7-O-glucoside (6); (B) kaempferol 3-O-feruloyldiglucoside-7-O-diglucoside (16); (C) quercetin 3-O-sinapoyltriglucoside-7-O-diglucoside (8). Figure S2: Negative ion electrospray MS2 mass spectra of hydroxycinnamoyl gentiobiosides: (A) 1,2-disinapoyl gentiobioside (24); (B) 1-sinapoyl-2-feruloyl gentiobioside (25); (C) 1,2-disinapoyl-2-feruloyl gentiobioside (28). Figure S3: Example of chromatograms acquired by HPLC-DAD (330 nm) after solid-phase extraction. See paragraph 3.4 for details. (A) Raw extract; (B) F50 fraction characterized by the predominance of flavonol-caffeoyl and kaempferol-sinapoyl derivatives; (C) F80 fraction characterized by the predominance of hydroxycinnamoyl gentiobiosides. For peak numbering, see Table 1. Figure S4: Scatter plot of measured vs. MLR prediction for DPPH scavenging activity, with root mean square error (RMSE), bias, and coefficient of determination (R2) in calibration (C, Cal) and cross-validation (CV). See paragraph 3.8. for details. Figure S5: Characteristics of the four *Brassica* wild species evaluated in the present work. Whole plant (left) and leaves (right) of *B. incana* (1-2), *B. macrocarpa* (3-4), *B. rupestris* (5-6), and *B. villosa* (7-8).
