# Supplementary material for: Phytochemical Characterization and In Vitro Antioxidant Properties of Four Brassica Wild Species from Italy
Source: Molecules. 2020 Jul 31;25(15):3495. doi: 10.3390/molecules25153495 (PMC7435896; doi:10.3390/molecules25153495)
Supplement: Supplementary file 1 [file molecules-25-03495-s001.pdf]

Supplementary Materials: Phytochemical Characterization and In Vitro Antioxidant Properties of Four *Brassica* Wild Species from Italy

Valentina Picchi [1,*], Roberto Lo Scalzo [1], Aldo Tava [2], Filippo Doria [3], Sergio Argento [4], Stefania Toscano [5], Simone Treccarichi [5] and Ferdinando Branca [5]

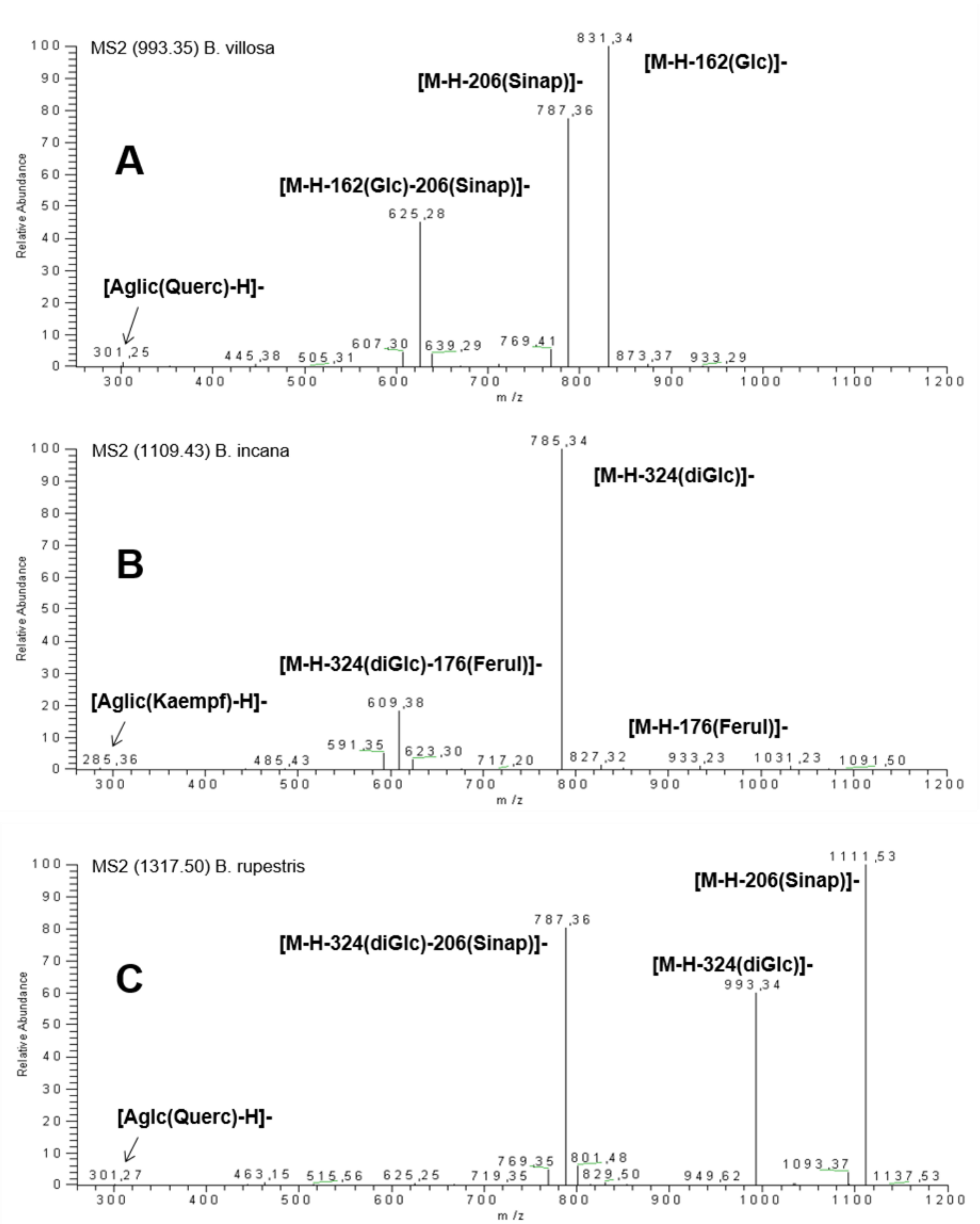

Figure S1. Negative ion electrospray MS^2 mass spectra of hydroxycynnamic acid esters of flavonol glycosides: **A**) quercetin 3-*O*-sinapoyldiglucoside-7-*O*-glucoside (**6**); **B**) kaempferol 3-*O*-feruloyldiglucoside-7-*O*-diglucoside (**16**); **C**) quercetin 3-*O*-sinapoyltriglucoside-7-*O*-diglucoside (**8**).

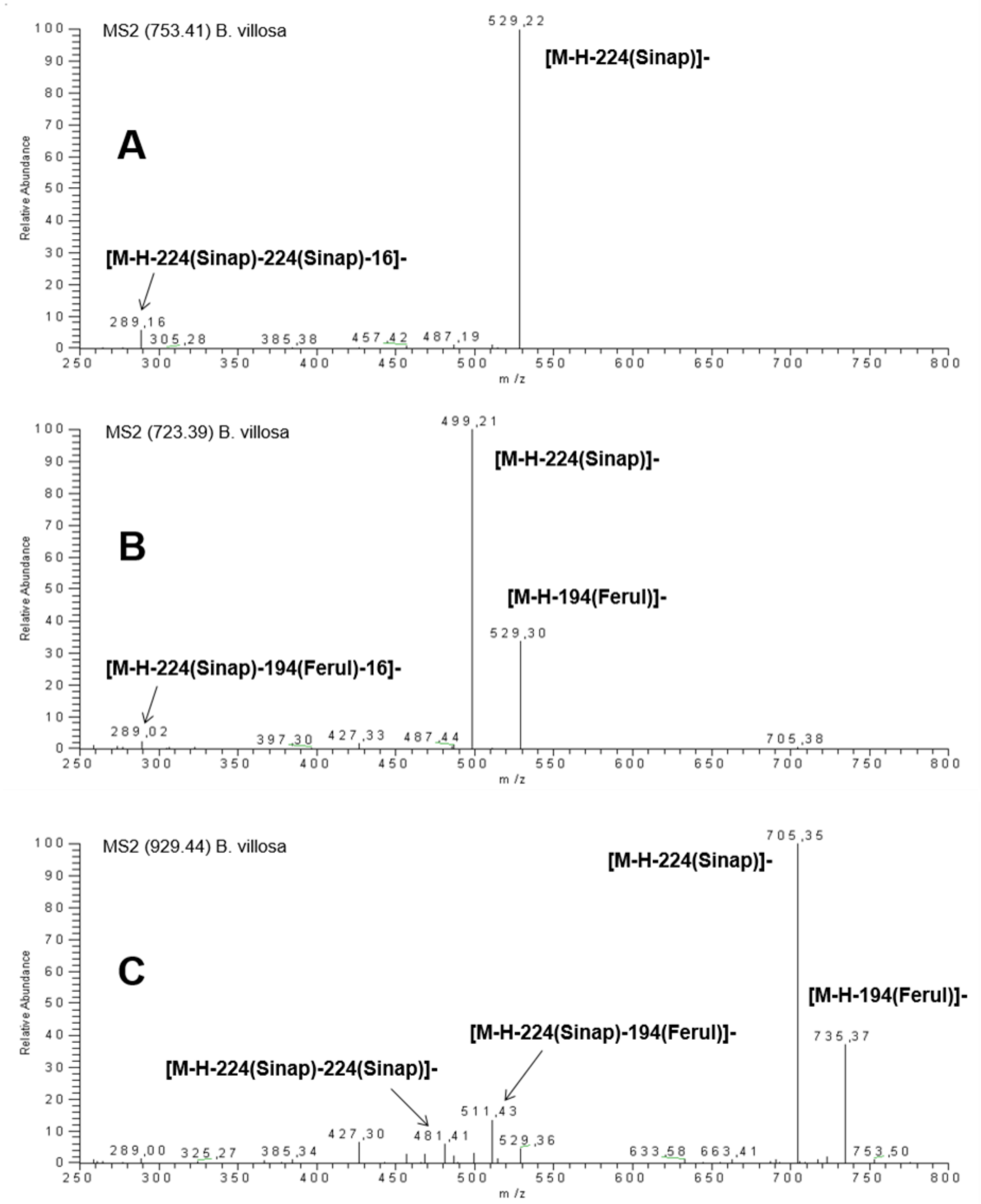

Figure S2. Negative ion electrospray MS^2 mass spectra of hydroxycinnamoyl gentiobiosides: **A**) 1,2-disinapoyl gentiobioside (**24**); **B**) 1-sinapoyl-2-feruloyl gentiobioside (**25**); **C**) 1,2-disinapoyl-2-feruloyl gentiobioside (**28**).

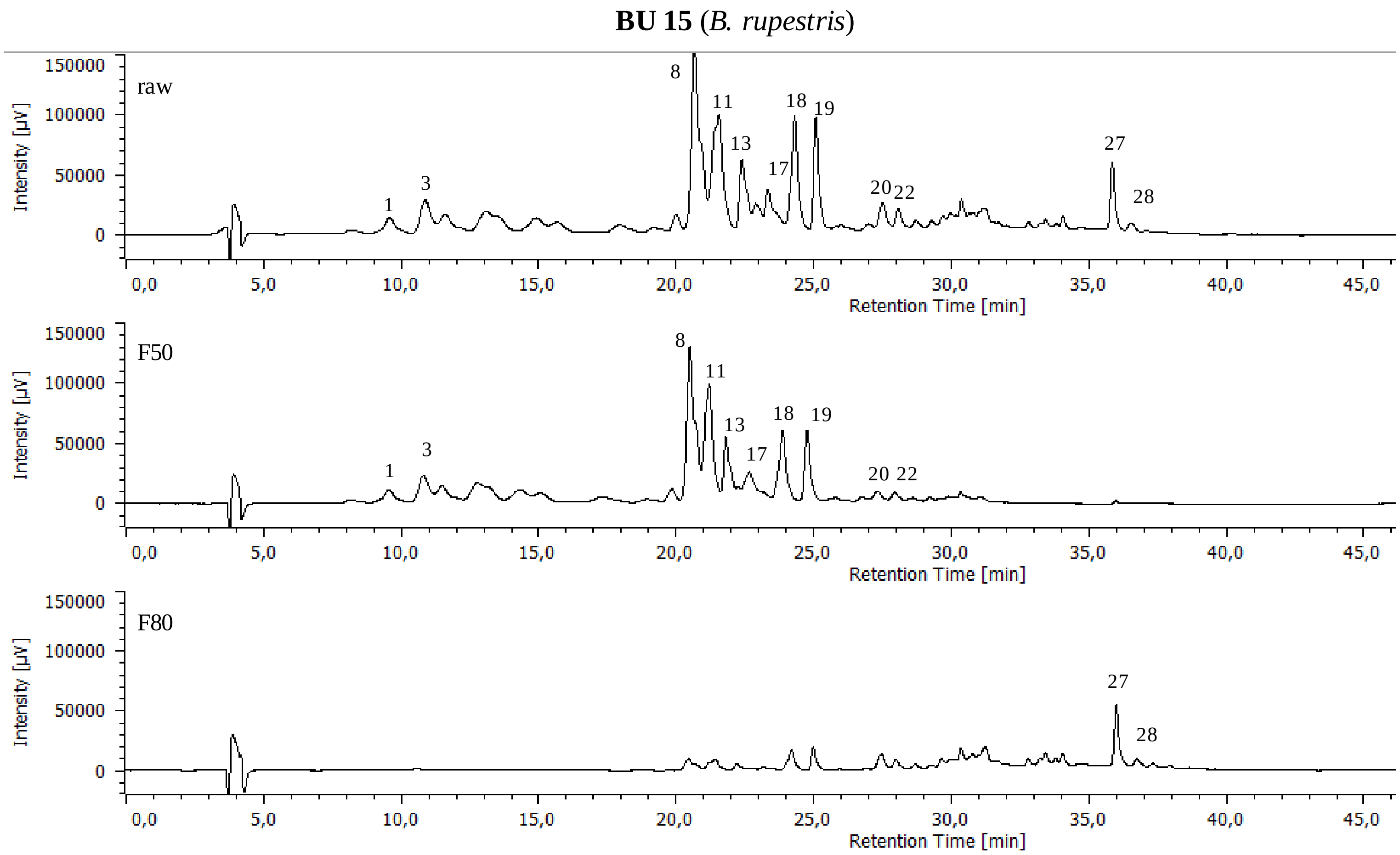

Figure S3. Example of chromatograms acquired by HPLC-DAD (330 nm) after solid-phase extraction. See paragraph 2.4 for details. (A) Raw extract; (B) F50, fraction characterized by the predominance of flavonol-caffeoyl derivatives and kaempferol-sinapoyl derivatives; (C) F80, fraction characterized by the predominance of hydroxycinnamoyl gentiobiosides. For peak numbering see Table 1.

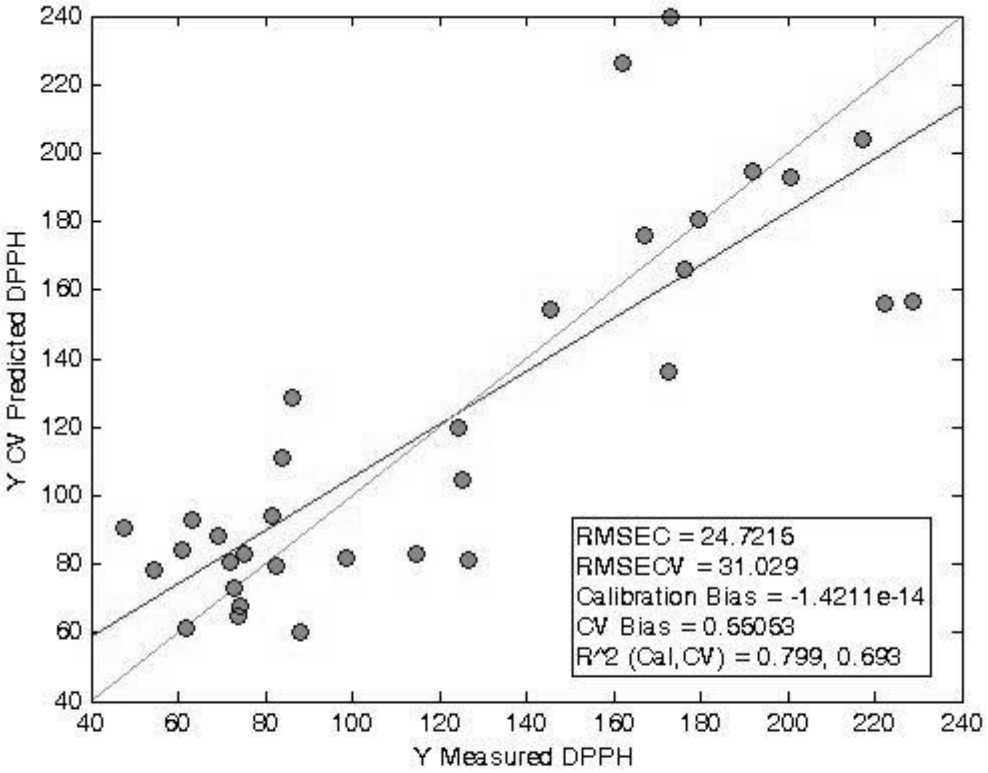

FigureS4. Scatter plot measured vs. MLR prediction for DPPH• scavenging activity, with root mean square error (RMSE), bias and coefficient of determination (R^2) in calibration (C and Cal) and in cross-validation (CV). See paragraph 2.8. for details.

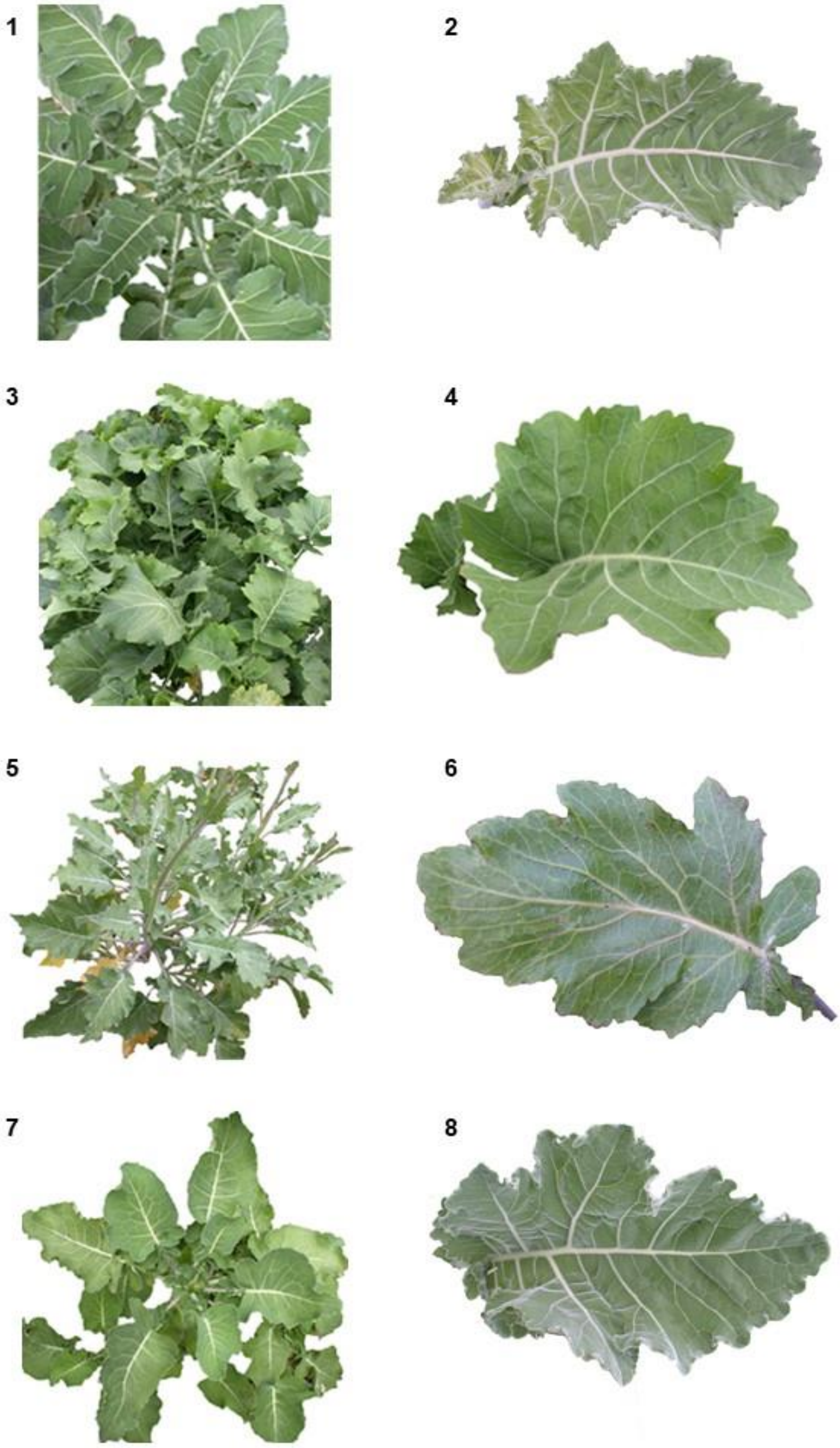

Figure S5. Characteristics of the four *Brassica* wild species evaluated in the present work. Whole plant (on the left) and particular of the leaves (on the right) of *B. incana* (1-2), *B. macrocarpa* (3-4), *B. rupestris* (5-6) and *B. villosa* (7-8).
